# Biomechanical Asymmetry of Strength and Dynamic Balance Kinetics in Middle-Ages with Adhesive Capsulitis of the Hip

**DOI:** 10.3390/ijerph192013093

**Published:** 2022-10-12

**Authors:** Moonyoung Choi, Yonghwan Kim

**Affiliations:** 1 Department of Sports Science Convergence, Dongguk University, Seoul 04620, Korea; 2 Department of Physical Education, Gangneung-Wonju National University, Gangneung 25457, Korea

**Keywords:** adhesive capsulitis of the hip, range of motion, strength, dynamic balance, hip joint, biomechanics, asymmetry, middle-ages

## Abstract

The representative clinical features of adhesive capsulitis of the hip (ACH) are restricted range of motion (ROM) and pain. However, reports on kinetics such as strength and dynamic balance that explain physical functions are rare. This study compared subjective hip scores using Copenhagen Hip and Groin Outcome Score (HAGOS) and ROM using a manual goniometer as well as strength using isokinetic equipment, and dynamic balance through the Y-balance test, between patients with ACH and healthy individuals. Data of 193 middle-aged participants (men: 99 and women: 94) were analyzed. The ACH group scored significantly lower in all six HAGOS sub-sections. Hip joint flexion, abduction, internal and external rotation ROM were significantly lower in ACH compared to healthy group. These results were the same for men and women. In the strength of men and women, flexion, adduction, and abduction, and dynamic balance in all three directions were significantly decreased in ACH. Meanwhile, there were no significant between-group differences in the functional decrease in extension and adduction of ROM, and extension strength. In conclusion, subjective evaluation and dynamic balance of patients with ACH were decreased in the all parts. In ROM, flexion, abduction, internal rotation, and external rotation were restricted except for extension and adduction. Men and women with ACH maintained extensor strength, but had weakened strength in flexion, adduction and abduction. This information will be useful for therapists to understand the biomechanical properties of ACH and to design effective rehabilitation programs.

## 1. Introduction

**Figure 1:**
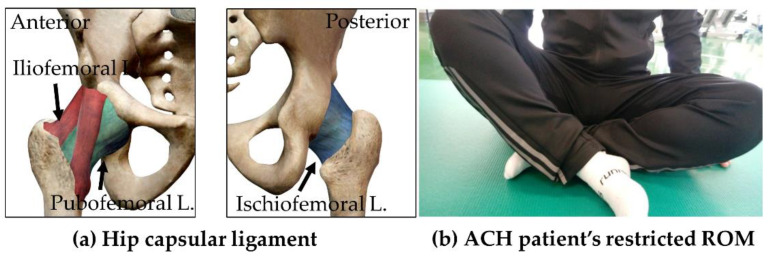
Hip capsular ligament anatomy and ACH patient’s characteristics.

Adhesive capsulitis is most common in the shoulder, but can occur in any joint and is often reported in the hip joint. Its incidence rate is approximately 2–5% in the general population [1]. The hip capsule comprises three external longitudinal ligaments (iliofemoral, pubofemoral, and ishciofemoral ligaments) that connect the acetabulum to the femur and internal fibers that run in the circumferential direction (Figure 1a) [2,3]. Adhesive hip capsulitis (ACH) is a condition characterized by fibrosis of the fibrous complex surrounding the hip joint. Although arthrofibrosis is a similar lesion, ACH is an intrinsic disorder that differs considerably from arthrofibrosis. ACH begins with synovial inflammation of the hip joint, and progresses to capsular fibrosis, whereas arthrofibrosis is scarring of the capsule and surrounding soft tissue caused by previous trauma or surgery, and is sometimes accompanied by serious structural disorders such as femoral impingement [4,5]

Primary or idiopathic ACH is of unknown etiology, and is more common in middle-aged women. The main symptoms of ACH include severe pain, restricted range of motion (ROM), and discomfort during physical activity (Figure 1b). Secondary ACH is presumably caused by prolonged stabilization and immobilization after trauma and surgery at other sites [6].

ACH remains under deliberation, as a standard diagnostic method has not yet been formulated. In general, a specialist makes the diagnosis by comprehensively considering the radiology results, medical history, physical examination, questionnaire, and counseling [7]. ACH comprises four stages: initial, freezing, frozen, and thawing stages. This process progresses naturally over a period of 5–18 months, with pain relief and improvement in ROM occurring during the final stage. Fortunately, ACH is not fatal and does not lead to permanent defects or other serious joint disease [8].

However, since severe pain causes discomfort and restrictions in activities of daily living (ADL), it is desirable to minimize the duration of this period, and treatment is administered to improve symptoms [4]. Conservative therapy, including rest, nonsteroidal anti-inflammatory drugs (NSAIDs), corticosteroid injections, and physical therapy is usually applied before resorting to surgical treatment. If there is no improvement after at least 3 months, radiology and arthroscopic synovectomy or capsulotomy pressure dilatation, open or arthroscopic synovectomy, lysis of adhesions, and capsular release may be considered. Nevertheless, experts recommend conservative therapy rather than surgery [9].

Among conservative therapies, exercise therapy can be managed by individual patients after learning the correct training method from a professional. As the representative symptom of ACH is restricted ROM, ROM restoration through stretching is recommended [10]. Stretching was taught to patients with ACH, followed by long-term follow-up to analyze compliance. ROM recovery was 44% after 9.8 months from the onset of symptoms and improved in over 80% of patients after 15 months [11]. In another case report, observation over a 2-year period revealed that ROM recovery exercises involving stretching and strength training were effective [8]. However, considering the characteristics of the hip joint with various movement directions, these studies have limitations in quantifying ROM because of the small number of participants, the large age range, and the mixed data of men and women. Moreover, although some studies have reported on ROM in ACH, few studies have analyzed the strength and dynamic balance required for stable movements, such as walking, running, climbing stairs, and changing direction.

Therefore, this study aimed to analyze the kinetic and functional characteristics of patients with ACH by comparing various ROM, muscle strength, and balance states between middle-aged men and women with ACH and healthy controls. This study is expected to provide basic data for use in devising effective rehabilitation programs, and to enhance kinetic understanding of ACH to therapists.

## 2. Materials and Methods

### 2.1. Experimental Design

The recruitment of participants in the ACH and healthy hip joint groups was advertised on bulletin boards of medical centers and public health centers. Participants voluntarily contacted the researchers and visited the hospital. The researchers explained all research procedures and obtained written consent from those who agreed to participate. This study was conducted in compliance with the Declaration of Helsinki and approved by the Institutional Review Board of Gangneung-Wonju University (approval number: GWNU IRB 2021-13).

The researchers investigated hip joint discomfort, as well as other joint medical and treatment histories. Specialists were involved in the analysis of patients diagnosed with ACH based on radiology results and physical examination. If ACH was diagnosed during treatment, the patient was informed about the study and the researchers provided details regarding the study. Selection and participation in the study were voluntary, and participants could opt-out at any time, with no penalty. The participants completed subjective hip joint evaluation using the Copenhagen Hip and Groin Outcome Score (HAGOS), ROM, isokinetic muscle strength, and dynamic balance using a Y-balance test (YBT) device. The test was performed on both sides, and the deficit values were calculated by comparing the healthy and ACH groups. The limb symmetry index (LSI) ROM, strength, and YBT were calculated by comparing both sides of each patient.

Deficit (%) = ((Healthy − Patient)/Healthy) × 100

ACH limb symmetry index (%) = (Involved side/Uninvolved side) × 100

Healthy limb symmetry index (%) = (Lower values side/Higher values side) × 100

### 2.2. Participants

The sample size was calculated using the G*power software (G*power 3.1, University of Düsseldorf, Düsseldorf, Germany), and the values presented in the program were applied; Wilcoxon-Mann-Whitney test (two groups); effect size d = 0.5; α error = 0.05; power, (1 − β err prob) = 0.80. The result is a sample size of 106.

**Figure 2:**
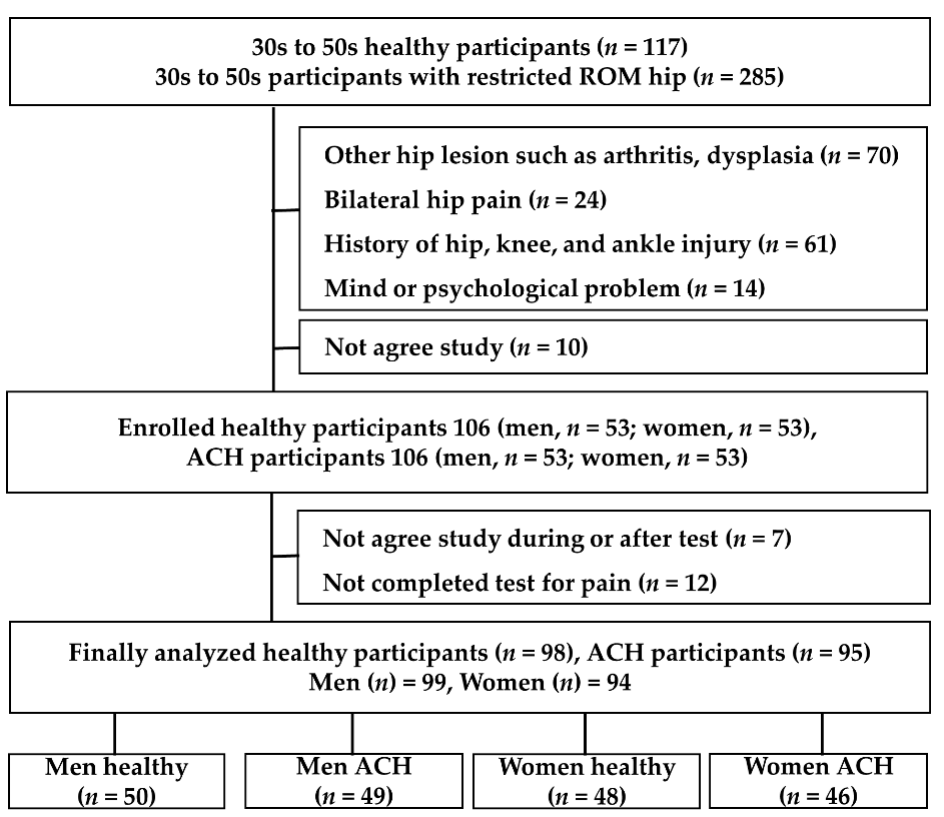
Participant’s exclusion and inclusion process.

The inclusion criteria were as follows: (1) men and women in their 30s to 50s, (2) no surgical history of hip, knee, ankle, or back surgery, (3) no history of severe disease. Moreover, medical history and hip pain status were investigated by consultation with the researcher, and patients with a history of surgery due to trauma or other diseases were excluded. The selected participants underwent a planned examination according to the study design, and patients with impingement syndrome, dysplasia, arthritis, and other pathological findings; patients with radiating pain due to low back pain and spinal-related diseases, and physical examination and physical examination because the pain was too severe in patients for whom kinematic examination was not possible, patients with bilateral pain, patients with complex pain in the ankle and knee, and patients taking medication for mental disorders were excluded. The healthy group had a visual analog scales (VAS) pain score of ≤ 3 and exhibited normal physical and radiology findings. The study was initiated with participants, and patients who requested to discontinue participation in the study due to pain, discomfort, change in decision to participate, or personal reasons were excluded. Therefore, final participants were 99 men (healthy controls: 50, ACH: 49) and 94 women (healthy controls: 48, ACH: 46) for the analysis (Figure 2).

#### 2.2.1. Copenhagen Hip and Groin Outcome Score Questionnaire

The HAGOS is a validated questionnaire with high reliability coefficients (intraclass correlation coefficients 0.82, 0.91). It was developed in 2011 to quantitatively identify hip joint problems, and is useful in clinical practice. The HAGOS consists of six sub-sections: pain, symptoms, ADL, sports and recreation, physical activity (PA), and quality of life (QoL) [12].

In this study, the survey was completed by the participant, and the researcher only assisted in cases of poor understanding of context, visual problems, or requests for help by the participant. The questionnaire was completed based on experiences during the week according to subjective pain. A 5-point Likert scale was used to select answers ranging from 0 to 4 points. Scores were calculated from 0 to 100 for each of the six subsections, with 100 representing excellent condition and 0 representing the most uncomfortable and severe symptoms.

#### 2.2.2. Range of Motion

**Figure 3:**
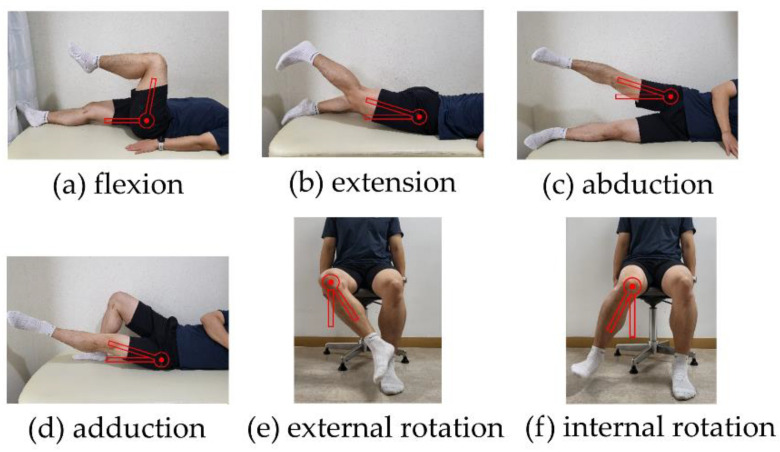
Hip range of motion measurement with goniometer.

Hip ROM was measured using a manual goniometer (Figure 3). Active ROM was measured, the compensatory action of the pelvis did not occur during measurement, and participants were instructed not to move the other leg and other body parts unnecessarily. The axis of the hip joint was the greater trochanter, and with the axis fixed, the participant performed the greatest possible ROM in the pain-free range. The maximum angle was measured slowly without recoil and then stopped for 2–3 s. Extension was measured with the participant in the prone position; internal and external rotation were measured with the sitting position in a chair; flexion in the supine position; and abduction and adduction in the side-lying position were evaluated. Both sides were measured; the non-painful side was measured first, and the painful side was subsequently measured. In the healthy group, the dominant side was measured first, followed by the non-dominant side. The higher values were used in the analyses.

#### 2.2.3. Y-Balance Test

Dynamic balance was measured using YBT equipment (Y Balance Test™, Cerder Park, TX, USA). In this test, the participant stood on one foot at the center of the Y-shaped equipment and pushed the plate as far away as possible with the opposite leg. This test is applicable for the elderly, athletes, and various patients requiring evaluation of physical function [13]. The healthy leg was measured first, followed by the affected leg. First, the anterior direction was measured, followed by the posteromedial and posterolateral directions. The test was performed barefoot for balance and stability.

To enhance participant understanding and increase familiarity with the test, the researcher first explained and demonstrated the procedure; the participant performed four practice exercises in each direction. After the practice was complete, each participant was given 5 min of recovery time, and three trials were performed in each direction. The highest value out of three successful executions of the procedure was recorded and analyzed.

#### 2.2.4. Hip Strength

For the hip joint strength test, flexion and extension, abduction, and adduction were measured using isokinetic equipment (Humac Norm CSMi; Stoughton, MA, USA) [14]. Isokinetic strength was measured in the concentric contraction mode at an angular velocity of 30°/s. To increase the understanding and familiarity of participants, the researcher provided explanations, demonstrations, and sufficient practice. The examination angles ranged from 0 to 100° for flexion and extension, and from 0 to 45° for abduction and adduction. If the participant’s restricted ROM was severe, it was actively performed to the extent possible. Practice was conducted at least three times, and after a 5 min rest, the actual test was conducted four times. First, extension and flexion were evaluated, and the participant adopted a supine position for the examination. The greater trochanter of the femur and the axis of the hip joint were aligned with the axis of the examination equipment. The pelvis and opposite leg were fixed with straps to stabilize the body. By so doing, we attempted to prevent excessive movement of other joints and body parts due to compensatory action. The equipment measured the weight of the legs and performed an automatic gravity correction; then the side-lying position was used during measurement of abduction and adduction; the axis of rotation was superior and medial to the greater trochanter of the femur. The maximum strength value was presented by the computer as torque (Nm), and for analysis, the torque value per body weight (Nm/kg) was calculated as a percentage.

### 2.3. Data Analysis

Statistical analysis of the collected data was performed using SPSS 25.0 (SPSS Inc., Chicago, IL, USA). Before performing the main analysis, a normality test was performed using the Kolmogorov-Smirnov test, which revealed that the main variables analyzed did not display normal distribution. Therefore, nonparametric analysis was performed to compare the two groups. Continuous variables were expressed as means and standard deviations, and the Mann-Whitney U test was used to compare the healthy and ACH groups. A history of shoulder adhesive capsulitis, dominant side, and affected side and the general characteristics were expressed in numbers and analyzed using the chi-square test. Statistical significance was set at *p* < 0.05.

## 3. Results

### 3.1. General Characteristics of Participants

Table 1 presents the general characteristics of the participants. There were no significant differences regarding age, height, weight, and body mass index (BMI) between the healthy and ACH groups. Additionally, there was no significant difference with respect to the dominant side and the history of shoulder adhesive capsulitis.

### 3.2. Copenhagen Hip and Groin Outcome Score Questionnaire

**Figure 4:**
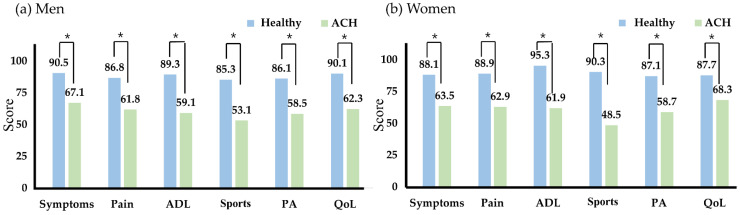
Subjective hip scoring with HAGOS. * *p* < 0.05; abbreviations: ACH, adhesive capsulitis of the hip; HAGOS, hip and groin outcome scale; ADL, activities of daily living; PA, physical activities; QoL, quality of life.

Figure 4 shows the results of the subjective hip score using HAGOS. Patients with ACH had significantly lower results in sub-sections (ADL, sports, and PA) than healthy group in men and women; pain, symptoms, quality of life, ADL, PA, and sports.

### 3.3. Range of Motion

Table 2 compares the ROM and LSI of the healthy group and the ACH group. In flexion, abduction, internal and external rotation, the ACH group exhibited significantly reduced ROM compared to the healthy group in both groups of men and women. There were no significant between-group differences in extension and adduction. The LSI in the healthy group was within ±10%, whereas the LSI in the ACH group was significantly lower in men external rotation (60.8%) and women (69.0%).

### 3.4. Measurement of Hip Strength Using Isokinetic Device

Table 3 shows the strength measured using isokinetic equipment. The ACH group of men and women had significantly lower strength in flexion, abduction, and adduction than the healthy group. Moreover, LSI showed the same result. This means that there was no difference in the strength of both hip joints in the healthy group, but in the ACH group, the strength of the affected side was lower than that of the healthy hip joint. Meanwhile, the extension strength and LSI in ACH of both sexes did not exhibit a significant decrease in strength compared to that in the healthy group, and LSI displayed a satisfactory value.

### 3.5. Dynamic Balance

Table 4 shows the dynamic balance results measured using YBT. In both men and women, ACH showed significantly lower values in all directions than the healthy group. Meanwhile, in the LSI, it was not significant in the posteromedial and posterolateral direction (The LSI are 89.8% to 91.2%). This means that the dynamic balance of the healthy side as well as the pain side of the ACH group was lowered along with it.

## 4. Discussion

In this study, we compared healthy controls and patients with ACH regarding ROM, muscle strength, and dynamic balance, evaluating the biomechanical characteristics of patients with ACH. The hip joint is a representative ball-and-socket joint, and has the advantage of being able to perform various movements as well as possessing a large ROM, and it plays a role in organically connecting the upper body and lower extremities. However, even a small loss in these functions can cause pain and discomfort in daily life [4,15].

Many clinicians have devised various questionnaires to screen for hip joint problems, and the evaluation results contributed not only to clinical diagnosis but also to designing therapeutic intervention strategies [16]. Among them, HAGOS is a questionnaire widely used in clinical practice as an evaluation tool to score subjective hip joint symptoms and functions detected in various environments such as sports, leisure, and physical activity as well as daily life [17]. As a result of this study, all six subsections of HAGOS were lower in the ACH group, and patients complained of greater discomfort, especially in physical activity and sports participation. As explained in previous studies [9,11], the typical symptom of ACH is pain, and moreover, the score may have been low because the hip joint was not smooth during activity due to limited ROM.

Evaluation of ROM in the clinic is the most common clinical parameter for diagnosing hip joint pathologies such as osteoarthritis, FAI, and ACH, and monitoring the effectiveness of treatment [18]. A lesioned hip results in significant functional limitations, including difficulties with ADL such as walking, wearing pants, sitting and standing, driving, tying shoelaces, and climbing stairs. Therefore, in orthopedic diseases, kinematic damage caused by ROM problems negatively affects quality of life [19].

As reported in several studies, the representative result of this study is the restricted ROM. Severe restriction occurred in movements requiring a large normal ROM (flexion, abduction, internal and external rotation); conversely, there was no significant difference regarding extension and adduction. These results are similar to those reported in previous studies [4,8]. Although the sample size was small for 3–4 people, small angles of internal rotation 20.0°, external rotation 17.5°, flexion 15.0°, and abduction 22.5° were shown [4]. In a case study, one participant reported significantly limited flexion abduction and external rotation, and the other participant reported decreased flexion, adduction, and internal rotation [8].

Hip joint ROM depends on the state of injury, morphological characteristics, and the surrounding soft tissue. The acetabulum of the pelvis, comprising the ilium, ischium, and pubis covers approximately 40% of the femoral head, covering the posterior part more than the anterior part [15]. Therefore, in the normal state, the extension ROM has a smaller angle compared to that of flexion, so there would be no significant difference from the healthy group. The presumed cause of flexion ROM limitation is that the posterior-inferior of the hip joint capsule is thin, while the anterior-superior is thick, fibrosis of the capsule occurs, and the tissue changes to a rigid state [6,15]. Since the ligaments constituting the joint capsule are formed longitudinally from the acetabulum to the femoral head, it is normal for the ligament length to increase slightly during rotation. However, the adhered tissue has limited movement owing to capsulitis during internal and external rotation [3].

An important characteristic of this study is that the muscle strength of patients with ACH was quantified using isokinetic equipment. Such studies are rarely found in the literature. The muscles of the hip joint act as a bridge that transfers the load of the upper body to the lower body, and it is the first joint that connects the tension generated from the axial skeleton to the lower body. Twenty-one muscles are associated with the hip joint, making it the joint with the most muscles in the human body [20]. It plays a key role in the dynamics that link daily movements, such as walking, stair climbing, and running to the femur, pelvis, spine, and knee [21]. Three main factors contribute to muscle strength. Muscle strength is proportional to muscle cross-sectional area and neuromuscular activity and inversely proportional to pain [22,23]. The significantly lower muscle strength in this study was probably due to an increase in pain rather than a decrease in muscle cross-sectional area. A previous study reported that painful areas in ACH occur in the groin, anterior, and lateral regions [8]. Therefore, in this study, there was no significant difference in muscle strength during extension, but a decrease in muscle strength during flexion, adduction, and abduction was observed. This could also be inferred from the HAGOS questionnaire. There was a greater difference in sports, PA, and ADL scores, with higher physical activity than constant symptoms and pain. A similar study conducted with soccer players revealed that HAGOS was significantly associated with hip strength [24].

Finally, we measured dynamic balance in patients with ACH. Dynamic balance plays a role in controlling posture when moving or changing the direction of the body [25]. Therefore, low dynamic balance in older individuals increases the risk of falls, which affects performance or causes impairment in activity for those involved in sports individuals [26,27,28]. Dynamic balance was low in this study, which was similar to the results of other studies showing a decrease in dynamic balance in patients with hip joint lesions [29,30,31]. Single-leg balance ability was reduced in patients with chondropathy, such as soft tissue injury of the hip joint and arthritis [31], and a study of the dynamic balance of patients with impingement syndrome, as in this study, also showed a significant decrease in the posteromedial and posterolateral directions than in the anterior direction [30]. In a study where dynamic balance was measured using YBT, the dynamic balance of asymptomatic or symptomatic patients with impingement syndrome was reduced equally [29]. The background of low dynamic balance in patients with ACH may be neuronal desensitization or sensory information abnormalities. Various nerve endings are distributed in the nasolabial labrum, which comprises the hip joint, detects pain, and plays a major role in proprioceptive feedback [32]. In addition, there are three nerve endings of proprioception in the joint capsule (type I Ruffini corpuscles, type II Pacinian corpuscles, and type IV free nerve endings). Han et al. [33] reported that type I and II proprioceptive organs did not exist in the surgically incised joint capsule, and a small number of type IV was observed in the pseudocapsule; however, they were significantly smaller than those of the normal joint capsule. Therefore, even if the joint capsule in this study was not in an incision state, the decreased receptor function of proprioceptive organs due to persistent capsulitis would have lowered dynamic balance function.

In this study, LSI showed the same results as the degree of ROM and Nm/kg of muscle strength, but there were different results only in YBT. LSIs of posteromedial and posterolateral were not significantly different from the LSI of the healthy group. In general, studies explain the normal range for LSI above 85% [34,35]. This means that although the healthy side of the ACH patient has normal ROM and muscle strength, the dynamic balance is lost along with the pain side. These results show results similar to those of previous study investigating the YBT of hip dysplasia patients. Even in the healthy side of the patient, the distance of the posterolateral direction was decreased compared to the healthy group [36], and patients with asymptomatic hip impingement syndrome showed significantly reduced distance in the YBT three directions compared to the healthy group [29].

The results of this study provide useful information that can be applied to the production of ACH rehabilitation programs. For the improvement of ROM in ACH patients, more attention should be paid to improvement of flexion and abduction rather than extension and addition. Likewise, muscle strength needs improvement in flexion, addition, and abduction. Lastly, as for the dynamic balance, posteromedial and posterolateral results were low compared to healthy participants in both men and women, there was no significant difference in LSI, so balance improvement training should be included for both sides.

The most common site of adhesive capsulitis is the shoulder, and the incidence in the hip joint is relatively low. The background of the low incidence may be because the symptoms of ACH are not as severe as those of the shoulder and, therefore, individuals affected do not visit the hospital [11]. The results of this study could enhance understanding of muscular strength and dynamic balance ability of patients with ACH by personnel treating ACH. Despite these findings, biomechanical results have limitations in terms of disease-specific explanation. This is because limited ROM and low muscle strength and balance are also found in individuals with other lesions, such as impingement syndrome and hip dysplasia [18,29,36]. Since our study was conducted at a single center, environmental diversity by region was not considered. In addition, the participants were middle-aged adults. Since this study included muscle strength and balance as variables, the older individuals who were highly likely to have various diseases and injuries were excluded. In addition, although functional results may differ depending on the participation rate in sports in daily life, they were not considered in this study. A limitation of this study was that the stage of pathological severity, which may be different for each individual, was not considered. It was previously reported that HAGOS improved when strength training was conducted in athletes [37]. A study demonstrated that improvement in ROM was positively impacted by strength training and active stretching in patients with ACH [11]. Future case-controlled studies should be conducted to identify effective therapeutic exercises by comparing various exercise interventions in men and women separating the sexes.

## 5. Conclusions

In middle-aged men and women with unilateral ACH, ROM was limited to flexion, abduction, internal and external rotation, and weakness was observed in flexion, adduction, and abduction. However, there was no significant functional decrease in the extension, adduction ROM, or extension muscle strength. Meanwhile, dynamic balance was reduced in all directions in men and women. Treatment practitioners should understand these biomechanical and kinetic characteristics and design specific rehabilitation training programs appropriate for ACH.

## Figures and Tables

**Table 1:** General characteristics of participants.

	Men			Women		
Variables	Healthy (*n* = 50)	ACH (*n* = 49)	*p*-Value	Healthy (*n* = 48)	ACH (*n* = 46)	*p*-Value
Age, years	49.1 ± 4.3	50.6 ± 4.5	0.229	48.1± 5.3	49.0 ± 5.5	0.524
Height, cm	171.5 ± 5.1	172.1 ± 5.9	0.219	162.2 ± 4.2	163.0 ± 4.5	0.398
Weight, kg	74.1 ± 4.6	73.9 ± 4.2	0.354	57.3 ± 5.6	58.9 ± 5.1	0.413
BMI, kg/m^2^	25.1 ± 2.0	24.9 ± 2.3	0.401	21.5 ± 1.3	21.7 ± 1.2	0.685
Dominant side (Left/Right), *n*	5/43	4/45	0.553	5/43	4/42	0.547
Shoulder or hip AC history, *n*	7	11	0.205	5	9	0.160
Pain side (Left/Right), *n*	–	27/22	–	–	22/24	–

*p* < 0.05; abbreviations: ACH, adhesive capsulitis of the hip; BMI, body mass index.

**Table 2:** Range of motion in healthy controls and patients with ACH.

	ROM, Degree				ROM, LSI (%)		
Variables	Healthy	ACH	Deficit, %	*p*-Value	Healthy	ACH	*p*-Value
Men							
Flexion	125.2 ± 9.8	103.4 ± 12.4	−17.6	<0.001 *	90.1	82.5	0.025 *
Extension	14.7 ± 3.5	13.6 ± 2.2	−7.5	0.254	95.9	90.2	0.189
Adduction	24.2 ± 5.9	23.1 ± 3.5	−4.5	0.316	97.5	89.6	0.153
Abduction	39.8 ± 8.2	28.3 ± 5.9	−28.9	<0.001 *	90.6	75.0	<0.001 *
Internal rotation	35.2 ± 6.2	21.6 ± 1.9	−38.6	<0.001 *	99.7	67.1	<0.001 *
External rotation	48.6 ± 6.8	41.8 ± 7.9	−14.0	<0.001 *	90.2	60.8	<0.001 *
Women							
Flexion	128.9 ± 11.3	98.3 ± 14.6	−23.2	0.010 *	90.9	81.6	0.002 *
Extension	15.8 ± 4.4	14.5 ± 4.1	−8.2	0.639	94.0	89.5	0.181
Adduction	25.3 ± 3.8	24.5 ± 5.1	−3.2	0.548	93.5	90.6	0.248
Abduction	42.1 ± 7.2	27.8 ± 2.9	−34.0	<0.001 *	90.6	71.7	<0.001 *
Internal rotation	42.3 ± 5.9	22.6 ± 5.1	−46.6	<0.001 *	90.4	69.0	<0.001 *
External rotation	45.3 ± 9.6	28.8 ± 7.8	−36.4	<0.001 *	91.2	75.3	<0.001 *

* p < 0.05; abbreviations: ACH, adhesive capsulitis of the hip; ROM, range of motion; LSI, limb symmetry index.

**Table 3:** Isokinetic strength of healthy controls and patients with ACH.

	Strength, Nm/kg				Strength, LSI (%)		
Variables	Healthy	ACH	Deficit, %	*p*-Value	Healthy	ACH	*p*-Value
Men							
Extension	2.80 ± 0.64	2.53 ± 0.45	−9.6	0.256	93.2	88.7	0.211
Flexion	2.16 ± 0.49	1.77 ± 0.34	−18.1	<0.001 *	94.1	75.2	<0.001 *
Adduction	1.75 ± 0.46	1.34 ± 0.42	−23.4	<0.001 *	94.5	68.6	<0.001 *
Abduction	1.61 ± 0.31	1.13 ± 0.28	−29.8	<0.001 *	96.3	74.1	<0.001 *
Women							
Extension	2.01 ± 0.46	1.87 ± 0.26	−7.0	0.301	92.9	89.8	0.281
Flexion	1.78 ± 0.31	1.35 ± 0.31	−21.9	<0.001 *	91.4	79.8	<0.001 *
Adduction	1.34 ± 0.44	1.09 ± 0.23	−18.7	<0.001 *	98.5	83.6	<0.001 *
Abduction	1.29 ± 0.39	1.01 ± 0.25	−21.7	<0.001 *	96.9	81.7	<0.001 *

* *p* < 0.05; abbreviations: ACH, adhesive capsulitis of the hip; LSI, limb symmetry index.

**Table 4:** Dynamic balance of healthy controls and patients with ACH.

	YBT, cm				YBT, LSI (%)		
Variables	Healthy	ACH	Deficit, %	*p*-Value	Healthy	ACH	*p*-Value
Men							
Anterior	70.2 ± 11.9	59.3 ± 13.6	−15.5	0.040 *	94.2	83.2	0.016 *
Posteromedial	98.3 ± 13.5	78.1 ± 15.1	−20.5	<0.001 *	92.6	90.6	0.105
Posterolateral	95.5 ± 14.4	76.3 ± 17.3	−20.1	<0.001 *	93.1	90.4	0.213
Women							
Anterior	62.3 ± 12.9	51.6 ± 15.6	−17.2	0.027 *	93.7	82.9	0.021 *
Posteromedial	77.1 ± 13.6	61.3 ± 16.1	−20.5	<0.001 *	92.5	90.2	0.071
Posterolateral	78.9 ± 10.3	60.9 ± 11.7	−22.8	<0.001 *	91.8	89.8	0.080

* *p* < 0.05; abbreviations: YBT, Y-balance test; ACH, adhesive capsulitis of the hip; LSI, limb symmetry index.

## Data Availability

The data are not publicly available because of privacy or ethics.
